# Epizootic Emergence of Usutu Virus in Wild and Captive Birds in Germany

**DOI:** 10.1371/journal.pone.0032604

**Published:** 2012-02-28

**Authors:** Norbert Becker, Hanna Jöst, Ute Ziegler, Martin Eiden, Dirk Höper, Petra Emmerich, Elisabeth Fichet-Calvet, Deborah U. Ehichioya, Christina Czajka, Martin Gabriel, Bernd Hoffmann, Martin Beer, Klara Tenner-Racz, Paul Racz, Stephan Günther, Michael Wink, Stefan Bosch, Armin Konrad, Martin Pfeffer, Martin H. Groschup, Jonas Schmidt-Chanasit

**Affiliations:** 1 German Mosquito Control Association (KABS), Waldsee, Germany; 2 Department of Zoology, Heidelberg University, Heidelberg, Germany; 3 Department of Virology, Bernhard Nocht Institute for Tropical Medicine, Hamburg, Germany; 4 Institute for Novel and Emerging Infectious Diseases, Friedrich-Loeffler-Institut, Greifswald, Germany; 5 Institute of Diagnostic Virology, Friedrich-Loeffler-Institut, Greifswald, Germany; 6 Department of Pathology, Bernhard Nocht Institute for Tropical Medicine, Hamburg, Germany; 7 Institute of Pharmacy and Molecular Biotechnology, Heidelberg University, Heidelberg, Germany; 8 Nature and Biodiversity Conservation Union (NABU) Baden-Württemberg, Stuttgart, Germany; 9 Avifauna-Nordbaden.de, Ornithologische Gesellschaft Baden-Württemberg (OGBW)-Nordbaden, Heidelberg, Germany; 10 Institute of Animal Hygiene and Veterinary Public Health, Faculty of Veterinary Medicine, University of Leipzig, Leipzig, Germany; University of Georgia, United States of America

## Abstract

This study aimed to identify the causative agent of mass mortality in wild and captive birds in southwest Germany and to gather insights into the phylogenetic relationship and spatial distribution of the pathogen. Since June 2011, 223 dead birds were collected and tested for the presence of viral pathogens. Usutu virus (USUV) RNA was detected by real-time RT-PCR in 86 birds representing 6 species. The virus was isolated in cell culture from the heart of 18 Blackbirds (*Turdus merula*). USUV-specific antigen was demonstrated by immunohistochemistry in brain, heart, liver, and lung of infected Blackbirds. The complete polyprotein coding sequence was obtained by deep sequencing of liver and spleen samples of a dead Blackbird from Mannheim (BH65/11-02-03). Phylogenetic analysis of the German USUV strain BH65/11-02-03 revealed a close relationship with strain Vienna that caused mass mortality among birds in Austria in 2001. Wild birds from lowland river valleys in southwest Germany were mainly affected by USUV, but also birds kept in aviaries. Our data suggest that after the initial detection of USUV in German mosquitoes in 2010, the virus spread in 2011 and caused epizootics among wild and captive birds in southwest Germany. The data also indicate an increased risk of USUV infections in humans in Germany.

## Introduction

Usutu virus (USUV) is an arthropod-borne, single-stranded RNA virus and belongs to the *Japanese encephalitis* virus group within the family *Flaviviridae*. USUV is closely related to West Nile virus (WNV), which killed thousands of birds in North America following its emergence in 1999. In 2001, USUV caused deaths in Blackbirds (*Turdus merula*) and Great Grey Owls (*Strix nebulosa*) in Austria [1], [2]. In the following years, USUV was demonstrated to circulate in several other European countries including Hungary, Switzerland, Spain and Italy [3], [4]. Neutralizing antibodies against USUV were also demonstrated in birds from the United Kingdom [5]. In contrast, no serological evidence for USUV circulation was found in birds from Germany between 2007 and 2011 [6], [7]. In 2009, two human cases with an USUV-related neuroinvasive illness were reported from Italy [8], [9]. In August 2010, USUV strain 1477 was isolated from a pool of *Culex pipiens pipiens* mosquitoes that were trapped in the city of Weinheim, southwest Germany [10]. In contrast, all mosquitoes trapped the year before (during 2009) in southwest Germany tested negative for USUV [10]. No obvious increase in the mortality of wild and captive birds was observed in southwest Germany in 2009 and 2010 [10].

Since June 2011, dead Blackbirds were frequently found around the cities of Mannheim and Heidelberg in southwest Germany. It was reported that Blackbirds showed signs of apathy, no escape behavior, staggered movements and ruffled plumage [11]. Moreover, it was reported that Blackbirds nearly disappeared in the cities of Mannheim and Heidelberg and neighboring areas in the northern valley of the Upper Rhine in southwest Germany. Therefore, it was suggested that the resident Blackbird populations were considerably decreased or almost extinct [11]. In addition, there were also reports on Blackbirds showing the typical symptoms of USUV-infection from outside the Upper Rhine-valley (Göppingen, Rems-Murr-Kreis, since early August 2011 [12]) or reports on their absence (Murrhardt, Rems-Murr-Kreis, August 2011 [13]; Bad Buchau (Federsee), Kreis Biberach, since July 2011 [14]). Moreover, considerable mortality of captive Great Grey Owls was reported from the zoological gardens of Heidelberg and Mannheim. According to published recommendations [15], [16], German ornithologists suggested that the mass mortality among Blackbirds could be related to USUV and started to collect data and dead birds.

## Results

**Figure 1:**
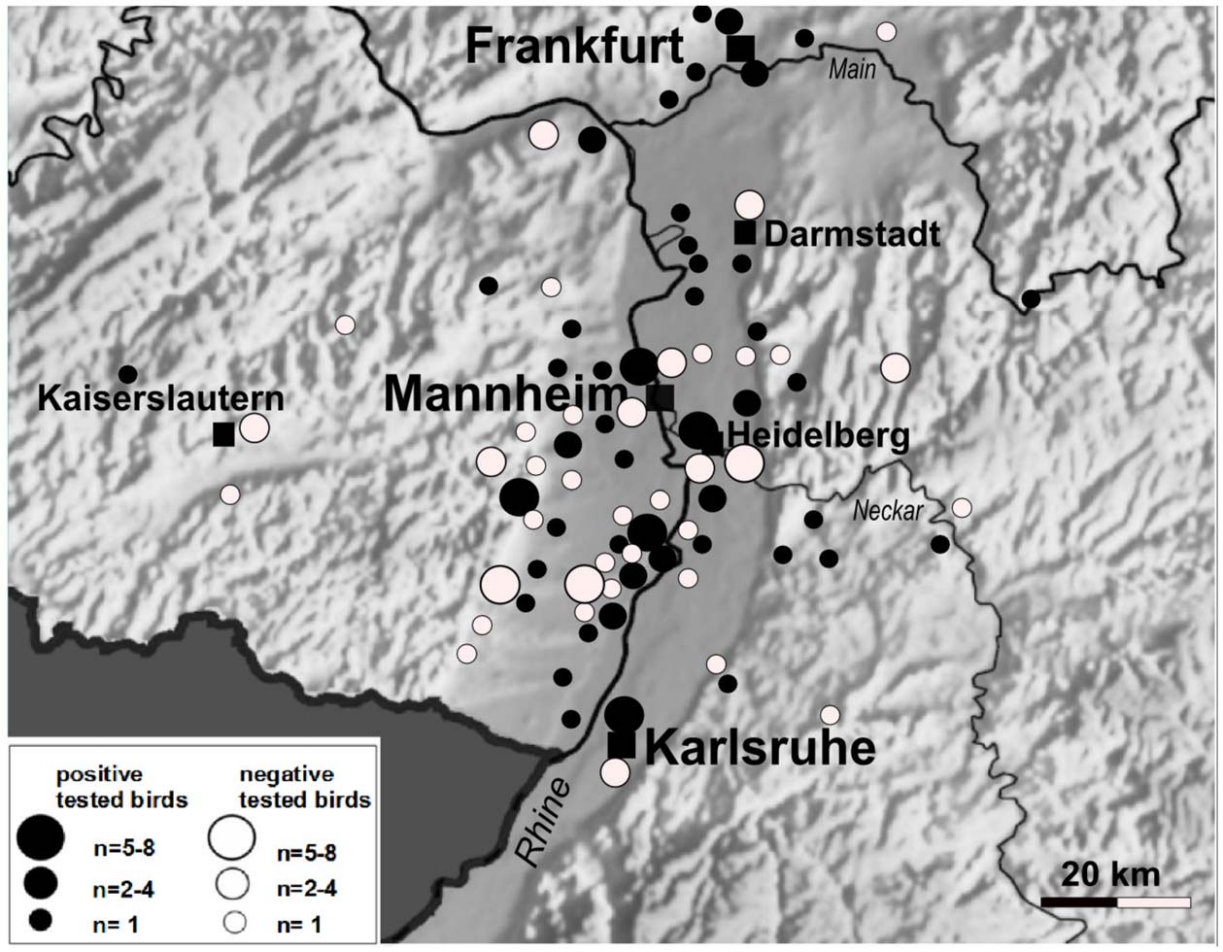
Spatial distribution of USUV RNA positive (n = 83) and negative (n = 61) tested birds found dead in southwest Germany. Black dot indicate birds tested positive for USUV RNA and white dot indicate USUV RNA negative birds. 79 birds were collected outside of region shown in the map.

**Figure 2:**
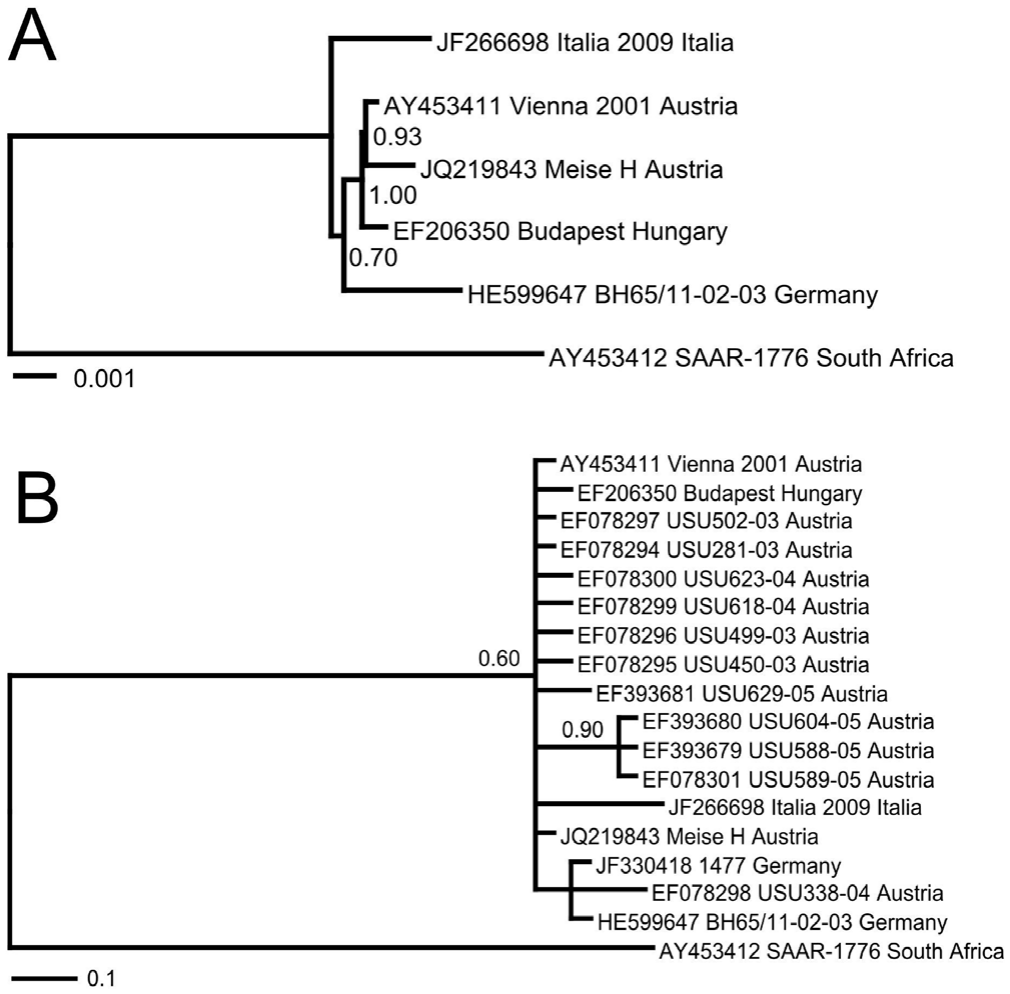
Phylogenetic analysis of the novel Usutu virus (USUV) strain BH65/11-02-03 detected in a dead Blackbird from Mannheim in southwest Germany. The phylogenetic trees inferred with MrBayes are based on nucleotide sequences and of USUV strains: **A** (length: 11003 nucleotides, complete polyprotein gene) and **B** (length: 1327 nucleotides, partial polyprotein gene). For each sequence, the GenBank accession number, strain designation, and strain origin are provided. Posterior probabilities higher than 0.5 are shown on each node. Scale bar indicates genetic distance in nucleotide substitutions per site.

From the beginning of June through November 2011, 223 birds (Table 1) were collected mainly in the upper Rhine valley (Fig. 1) and sent to the Bernhard Nocht Institute for Tropical Medicine and the Friedrich-Loeffler-Institut for USUV diagnostics. USUV-specific RNA was detected in the organs of 86 animals from 6 species using USUV-specific real-time RT-PCR (Table 1). The cycle threshold (ct) values were in the range of 11–20 for all organs tested (brain, liver, spleen, heart and lung), demonstrating pantropism with high virus load in tissue. The real-time RT-PCR data were confirmed by amplification of short fragments using a one-step pan-flavivirus RT-PCR [17] and sequencing of the PCR fragments. To rapidly generate comprehensive sequence information, we employed deep sequencing of the cDNA libraries generated from RNA of organs of a dead Blackbird from Mannheim (no. BH65/11-02-03). This approach yielded 457,835 reads for the spleen sample and 342,644 reads for the liver sample. Of these, 3798 reads (0.83%) (spleen) and 1612 reads (0.47%) (liver), respectively, represented USUV sequences. Assembly of the raw data generated identical nearly full-length genomic sequences from both libraries. The newly sequenced genome of USUV strain BH65/11-02-03 comprised a single open reading frame coding for the complete USUV polyprotein with 3434 amino acids. The sequence was submitted to GenBank and assigned the accession number HE599647. The genetic distance calculated with the complete nucleotide sequence of polyprotein between strain BH65/11-02-03 and the other European USUV strains Vienna 2001, Meise H from 2002 (the sequence was also generated in this study from the virus isolate, see below), and Budapest from 2005 is ≥99%. At the amino acid level, strains BH65/11-02-03 and Vienna 2001 were almost identical (99.7%; 10 amino acid differences). Hence, the vast majority of the mutations were silent. In agreement with the distance analysis, phylogenetic reconstruction using complete nucleotide sequences of polyprotein revealed a close relationship of strain BH65/11-02-03 with the European but not the African USUV strains (Fig. 2A). When considering also partial sequences in the phylogeny, strain BH65/11-02-03 is most closely related to USUV strain 1477 with 100% identity at nucleotide level (1327 nucleotide fragment) (Fig. 2B). Strain 1477 had been isolated from a pool of *Culex pipiens pipiens* mosquitoes trapped in 2010 in southwest Germany [10]. USUV strain 1477 is closely related with but not identical to the USU338-04 strain from Austria, which was detected in a dead Blackbird in 2004, as well as to the full-length sequences mentioned above.

**Table 1:** Number of dead birds analyzed for Usutu virus infection by real-time RT-PCR (time period June to November 2011).

Order	Common name	Scientific name	Migration pattern*	Housing	USUV RNA positive/tested (% positive)
Passeriformes	Blackbird	*Turdus merula*	R, P	Wild	72/148 (48%)
	Common Starling	*Sturnus vulgaris*	R, P, S	Wild	3/17 (17%)
	Canary	*Serinus canaria domestica*	-	Captive	2/5 (40%)
	House Sparrow	*Passer domesticus*	R	Wild	1/1 (100%)
	European Greenfinch	*Carduelis chloris*	R, P, M	Wild	0/10
	Fieldfare	*Turdus pilaris*	S	Wild	0/6
	Blackcap	*Sylvia atricapilla*	M	Wild	0/6
	Song Thrush	*Turdus philomelos*	M	Wild	0/6
	European Robin	*Erithacus rubecula*	P	Wild	0/4
	Eurasian Jay	*Garrulus glandarius*	R, P	Wild	0/3
	Blue Tit	*Parus caeruleus*	R	Wild	0/2
	Yellowhammer	*Emberiza citrinella*	R, P, S	Wild	0/1
	Carrion Crow	*Corvus corone*	R, P	Wild	0/1
	Nightingale	*Luscinia megarhynchos*	L	Wild	0/1
Galliformes	Grey Partridge	*Perdix perdix*	R	Wild	0/2
Piciformes	Green Woodpecker	*Picus viridis*	R	Wiild	0/1
Strigiformes	Great Grey Owl	*Strix nebulosa*	-	Captive	6/6 (100%)
Coraciiformes	Common Kingfisher	*Alcedo atthis*	R, P, M	Wild	2/2 (100%)
Sphenisciformes	Humboldt Penguin	*Spheniscus humboldti*	-	Captive	0/1
TOTAL					86/223 (38%)

*R = resident, P = partial, S = short distance, M = migratory, L = long distance.

**Figure 3:**
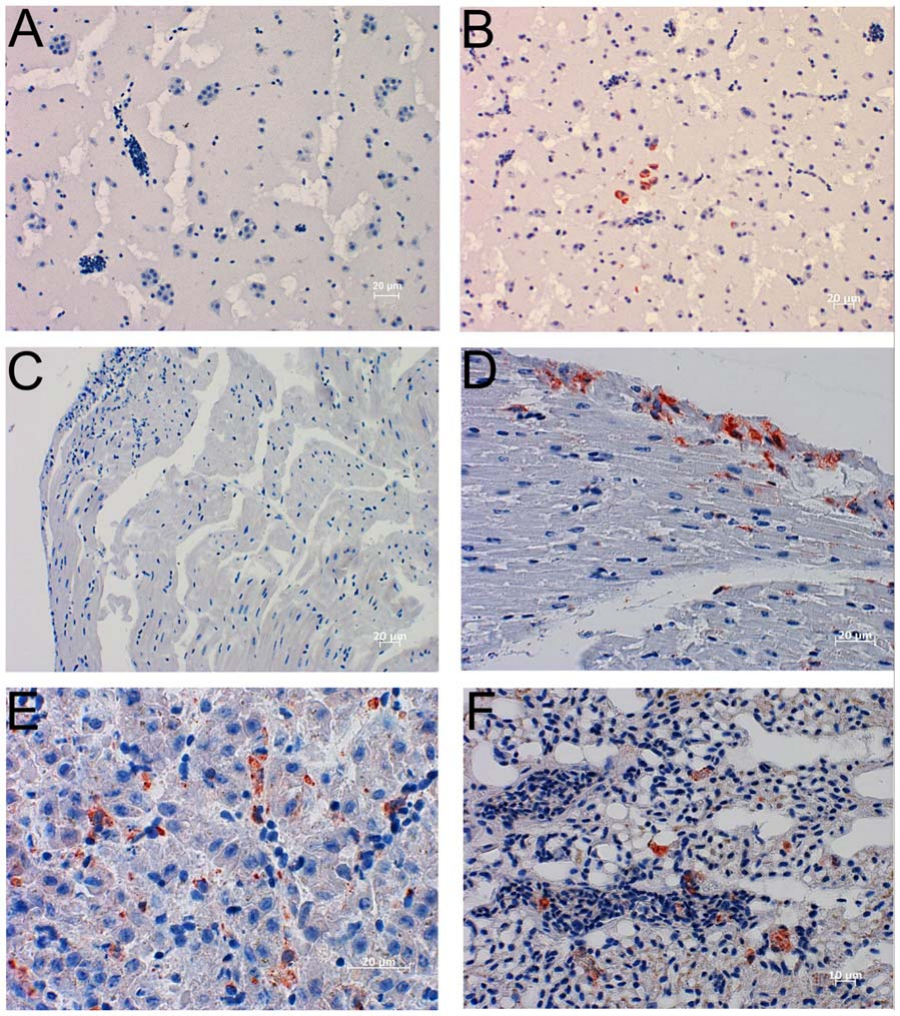
Immunohistochemistry (IHC) of USUV-uninfected (A, C) and –infected (B, D, F and F) blackbird organs using an USUV-specific murine monoclonal antibody. (A) USUV-uninfected blackbird brain. (B) USUV-infected blackbird brain showing a group of USUV-positive neurons (in red). (C) USUV-uninfected blackbird heart. (D) USUV-infected blackbird heart, USUV-positive cells are localized in the endocardium (in red). (E) USUV-infected blackbird liver, disseminated USUV-positive Kupffer cells (in red). (F) USUV-infected blackbird lung, disseminated USUV-positive cells (in red).

USUV was isolated in cell culture from 18 of 38 USUV RNA-positive Blackbirds. All isolated USUV strains caused CPE in Vero cells and virus growth was monitored by detection of USUV-specific RNA in the supernatant of the infected cell cultures after five passages using real-time RT-PCR. In addition, USUV strain Meise H was isolated from the brain of a Blue Tit which was found dead at a cemetery in Baden, Austria, on August 29th 2002. The complete genome of this isolate was sequenced and included in the phylogenetic analysis described above. Organs from the remaining USUV RNA-positive birds were not used for virus isolation because of autolysis. Electron microscopy of infected cell cultures showed enveloped viral particles ∼60 nm in diameter (data not shown). Immunohistochemistry (IHC) of brain, heart, liver, and lung of USUV RNA-positive blackbirds was performed using a recently developed USUV-specific monoclonal antibody (P. Emmerich, unpublished). USUV antigen was detected in all organs of infected birds demonstrating widespread virus replication in various tissues. Virus was specifically found in brain neurons and hepatic Kupffer cells, consistent with previous reports [18] (Figure 3 B, D, E and F). Antigen was not detected in USUV RNA-negative control birds (Figure 3 A and C).

## Discussion

This paper describes the first epizootic emergence of USUV in birds in Germany. The infection was diagnosed by real-time RT-PCR, deep sequencing of tissue RNA, virus isolation, and immunohistochemistry. Real-time RT-PCR and immunohistochemistry data demonstrated a high level of virus replication in various organs and tissues (pantropism), which presumably played a role in the fatal outcome of the infection in these birds. In agreement with previous studies [1], [2], Blackbirds, House Sparrows, and Great Grey Owls were shown to be predominantly susceptible for USUV. In addition, our study demonstrates the susceptibility of Canary, Common Starling, and Common Kingfisher for USUV infection. We have confirmed, that birds of the order Passeriformes and Strigiformes are mainly affected by USUV, which is in line with observations from Austria [19]. In addition, our observations on the spatial distribution of USUV were confirmed by a Citizen Science Program of NABU for the survey of winter bird populations in Germany in January 2012. When compared to January 2011, a statistically significant decrease of the Blackbird population was observed in the upper Rhine valley but not in other parts of Germany (NABU website. Available: http://www.nabu.de/aktionenundprojekte/stundederwintervoegel/ergebnis/?jahr=2012&ort=&vogelart=Amsel&bundesland=. Accessed 2012 Feb 4.). Although the birdwatcher scene was informed and alerted [10]–[15], it might well be that the bird species collection was biased in a way that very common and abundant (House Sparrow, Tit, Blackbird, and Common Starlings) or flamboyant bird species (Blackbird, Common Kingfisher) were more frequently tested than other bird species, which may have died unnoticed. Our observations also do not exclude that birds of other species may be infected but do not die from the infection.

The known resident or partial migration pattern of USUV-positive bird species and the phylogenetic analysis suggest that the epizootic USUV strain most likely spread from Austria or a neighboring county to Germany. An independent introduction from Africa to Germany is not supported by our data. Interestingly, the USUV strains in Germany, Austria, Switzerland, and Hungary are nearly identical at the nucleotide level indicating a high genetic stability of the virus over the last 10 years.

USUV was found in Germany in a mosquito-based surveillance program for arboviruses already one year before the epizootic [10]. This pre-epizootic discovery demonstrates the importance and forecasting power of such monitoring programs. The specific conditions which facilitated virus emergence in Germany are speculative. Retrospective analysis of environmental factors during the USUV epizootics in Austria disclosed an influence on USUV dynamics of climatic factors affecting the mosquito population [20]. In addition to supply of water, temperature plays a decisive role in mosquito development and influences the progression of generations and the size of the vector populations [21], [22]. The higher average temperatures in April and May 2011 compared to the average temperatures of the years 1961 to 1990 (>2 K and >4 K, respectively) led to an increase in the number of Culex generations (N. Becker, unpublished data) and may have contributed to the rapid spread of USUV in southwest Germany. This is in agreement with the Austrian model from which it was deduced that the high number of USUV cases in 2003 was due to the early beginning of the extraordinary hot summer in that year [20]. Detailed analyses of environmental conditions in Germany during emergence of the virus may strengthen the scientific basis of these models and increase the reliability of future predictions.

When USUV first emerged in Austria, it was uncertain if the virus was able to establish endemicity in our climate [1]. Ten years later the virus is still circulating in Austria, but bird mortality declined dramatically. One possible explanation for this phenomenon is the development of herd immunity over the years providing a certain level of protection to the bird population [23]. Monitoring of bird deaths as well as seroprevalence studies in wild and captive birds have to be conducted in Germany to see whether the virus is further spreading and/or whether the incidence of infections decreases with an increasing immunity in birds. USUV is pathogenic to humans, though cases seem to be very rare [8], [9]. This might be related to the fact that USUV-infected mosquitoes are mainly ornithophilic and bite humans rarely. However, the large number of infected birds in an epizootic may facilitate the transmission of the virus to other mosquito species which are more anthropophilic. Public health authorities and clinicians in Germany should be aware of the risk of USUV infection in humans and consider this virus in cases of meningoencephalitis.

## Materials and Methods

### Ethics Statement

No specific permits were required for the collection of dead birds in Germany.

### Dead bird collection

Since June 2011, ornithologists, entomologists and virologists alerted the general public by mass media reports about the mass mortality of birds in southwest Germany. People who found dead birds in Germany were requested to inform the German Mosquito Control Association (KABS), Nature and Biodiversity Conservation Union (NABU) or Bernhard Nocht Institute for Tropical Medicine (BNI). The information's were registered online by NABU (NABU website. Available: https://www.nabu.de/tiereundpflanzen/voegel/forschung/14160.html. Accessed 2012 Feb 4.) and dead birds were directly submitted by the people to BNI or Friedrich-Loeffler-Institut (FLI) or collected from the people by assistants of KABS. Strongly autolyzed birds were not further processed.

### Real-time RT-PCR

Birds were dissected and viral DNA and RNA were extracted from brain, liver, or spleen using the RTP DNA/RNA Virus Mini Kit according to the instructions of the manufacturer (Invitek, Berlin, Germany). Extracted RNA was analyzed by using an USUV-specific real-time reverse transcription–polymerase chain reaction (RT-PCR) with primers USUTU F (5′-CGTTCTCGACTTTGACTA-3′, nucleotide positions 3294–3311; nucleotide positions are given according to numbering in USUV strain Vienna, GenBank accession no. AY453411), USUTU R (5′-GCTAGTAGTAGTTCTTATGGA-3′, nucleotide positions 3384–3364), probe USUTU P (5′-FAM-ACCGTCACAATCACTGAAGCATBHQ1-3′, nucleotide positions 3325–3346, FAM, 6-carboxyfluorescein; BHQ-1, black hole quencher 1). The target was a 91-bp-long region of the nonstructural protein 1 gene. Real-time RT-PCR was used as described [10].

### Pan-flavivirus RT-PCR

Samples tested positive for USUV RNA with the real-time RT-PCR described above were tested with a one-step pan-flavivirus RT-PCR modified from [17] to confirm the results. Only the primers mFU1 (5′ TACAACATGATGGGAAAGCGAGAGAAAAA-3′) and CFD2 (5′-GTGTCCCAGCCGGCGGTGTCATCAGC-3′) were used with the One-step RT-PCR Kit according to the instructions of the manufacturer (Qiagen, Hilden, Germany). Thermal cycling conditions were comprised of an initial RT step at 50°C for 30 min, followed by 95°C for 15 min and followed by 45 cycles of denaturation at 95°C for 20 s, annealing at primer pair-specific temperatures for 20 s, and polymerization at 72°C for 20 s. The PCR product (∼250 bp) was visualized on a UV transilluminator following separation on 2.5% agarose gels containing ethidium bromide.

### Full-length sequencing using the Genome Sequencer FLX

For determination of the genomic sequence of USUV strain BH65/11-02-03, total RNA was extracted from liver and spleen from a dead Blackbird from Mannheim using Trizol (Invitrogen, Darmstadt, Germany) in combination with RNeasy Mini Spin Columns (Qiagen, Hilden, Germany) was used as input for preparation of a 454 Genome Sequencer FLX (Roche, Mannheim, Germany) sequencing library following the manufacturer's instructions using rapid library adaptors. Before library preparation, the content of target and host RNA molecules was assessed by RT-qPCR. Those samples with the highest ratio of USUV RNA compared to 18 s rRNA were selected for sequencing. The resulting libraries were quantified by qPCR with a KAPA Library Quant Roche 454 Titanium kit (KAPA Biosystems, Cape Town, South Africa) and were used as input for the emulsion PCR with 0.08 copies per bead. The beads were sequenced in one region of a PTP with the GS FLX Titanium chemistry. The resultant reads were assembled using the 454 assembler software newbler v2.6 (Roche). The genomic sequence of USUV isolate Meise H was determined by conventional RT-PCR using primer pairs to generate overlapping fragments of 1200–1800 nucleotides in length and subsequent cycle sequencing in an ABI PRISM 3130 Genetic Analyzer (Applied Biosystems).

### Immunohistochemical analysis

Tissue samples were fixed in 7% buffered formalin. After embedding in paraffin wax, sections were stained with haematoxylin and eosin and Giemsa stain for routine histology. For immunohistochemistry, paraffin sections were dewaxed and placed in a domestic pressure cooker containing citrate buffer (pH 6) and boiled for 2 minutes to block the endogenous peroxidase, the sections were treated with peroxidase blocking reagent (DakoCytomation, Hamburg, Germany) for 20 min. After applying the USUV-specific murine monoclonal antibody 4E9 (P. Emmerich, unpublished) (1∶100 for 60 min), the sections were processed according to the manufacturer's instructions for the following kits: DCS-DetectionLine (PD999RP), Universal Block (UL123R999), diaminobenzidine tetrahydrochloride-substrate kit (DC137C999) all by Innovative Diagnostik-Systeme (Hamburg, Germany). The sections were counterstained with haematoxylin, mounted and examined using an AxioIimager M1 microscope (Carl Zeiss, Jena, Germany). USUV strain 1477-infected Vero E6 cells (ATCC CRL-1586) served as positive control.

### Virus isolation

Virus isolation was done with well-preserved heart specimen from 38 USUV infected Blackbirds. About 8 mm of heart was homogenized in 1 ml cell culture medium using a bead mill. The homogenate was cleared by centrifugation and a confluent African green monkey kidney (Vero E6) cell monolayer in a 25-cm^2^ cell-culture flask was inoculated with 100 µl homogenate. Cells were incubated for 1 h at 37°C, and after this, 10 ml of cell culture medium was added and the cells were incubated at 37°C in the presence of 5% CO_2_ for 1 week and observed daily for evidence of cytopathology. Cell cultures showing a cytopathic effect (CPE) were passaged. Virus growth was monitored by detection of USUV-specific RNA in the supernatant of the infected cell cultures after five passages using real-time RT-PCR.

### Phylogenetic analysis

Phylogenetic analysis included the novel USUV complete coding regions sequences from a dead Blackbird from Mannheim (stain BH65/11-02-03, Germany) and a dead Blue Tit from Baden (strain Meise H, Austria) as well as USUV complete coding regions sequences available from GenBank by December 2011. Nucleotide sequences were aligned by using BIOEDIT sequence editing program [24]. FindModel (http://www.hiv.lanl.gov/content/sequence/findmodel/findmodel.html) identified the general time-reversible model of sequence evolution with a gamma distribution of among-site nucleotide substitution rate variation (GTR+gamma) as the substitution model that best describes the data in the alignments. The phylogeny was inferred by the Bayesian Markov Chain Monte Carlo (MCMC) method implemented in MrBayes 3.0 software [25] using nucleotide sequence alignments of the complete USUV coding regions (6 taxa, 11003 sites). Three heated chains and a single cold chain were used in all MCMC analyses, which were run for 1,000,000 generations, sampling one tree every 100 generations. Trees obtained before convergent and stable likelihood values were discarded. Four independent runs, each started from different, randomly chosen trees, were performed to assess convergence. Posterior probabilities for nodes were assembled from all post burn-in trees.
