# Correction: Epizootic Emergence of Usutu Virus in Wild and Captive Birds in Germany

**DOI:** 10.1371/annotation/6841c4e1-58e6-4412-9b71-bd6bc8bbe549

**Published:** 2012-08-27

**Authors:** Norbert Becker, Hanna Jöst, Ute Ziegler, Martin Eiden, Dirk Höper, Petra Emmerich, Elisabeth Fichet-Calvet, Deborah U. Ehichioya, Christina Czajka, Martin Gabriel, Bernd Hoffmann, Martin Beer, Klara Tenner-Racz, Paul Racz, Stephan Günther, Michael Wink, Stefan Bosch, Armin Konrad, Martin Pfeffer, Martin H. Groschup, Jonas Schmidt-Chanasit

The following information was missing from the Funding section: The study was also supported by the European Union FP7 project European Management Platform for Emerging and Reemerging Infectious Disease Entities (EMPERIE; no. 223498). 

